# Myocardial Strain Measurements Obtained with Fast-Strain-Encoded Cardiac Magnetic Resonance for the Risk Prediction and Early Detection of Chemotherapy-Related Cardiotoxicity Compared to Left Ventricular Ejection Fraction

**DOI:** 10.3390/diagnostics15151948

**Published:** 2025-08-03

**Authors:** Daniel Lenihan, James Whayne, Farouk Osman, Rafael Rivero, Moritz Montenbruck, Arne Kristian Schwarz, Sebastian Kelle, Pia Wülfing, Susan Dent, Florian Andre, Norbert Frey, Grigorios Korosoglou, Henning Steen

**Affiliations:** 1 Cardio-Oncology Center of Excellence, Saint Francis Healthcare, Cape Girardeau, MO 63703, USA; 2 Myocardial Solutions Incorporated, Morrisville, NC 27599, USA; jim.whayne@myocardialsolutions.com (J.W.); farouk.osman@myocardialsolutions.com (F.O.); rafael.rivero@myocardialsolutions.com (R.R.); 3 Department of Cardiac Imaging (MRI), Center for Preventive Medicine, Marienkrankenhaus Hamburg, 22087 Hamburg, Germany; montenbruck.zpm@marienkrankenhaus.org (M.M.);; 4 Cardiac Imaging Unit, Deutsches Herzzentrum der Charité, 10117 Berlin, Germany; sebastian.kelle@dhzc-charite.de; 5 Department of Cardiology, Angiology, and Intensive Care Medicine, Deutsches Herzzentrum der Charité, 10117 Berlin, Germany; 6 Breast Center, Jerusalem Hospital, 20357 Hamburg, Germany; pia@sidekickhealth.com; 7 University of Rochester, Rochester, NY 14627, USA; susan_dent@urmc.rochester.edu; 8 Department of Cardiology, Angiology, and Pneumology, Heidelberg University, 69120 Heidelberg, Germany; florian.andre@med.uni-heidelberg.de (F.A.); norbert.frey@med.uni-heidelberg.de (N.F.); 9 GRN Academic Teaching Hospital Weinheim, 69469 Weinheim, Germany; gkorosoglou@hotmail.com; 10 Cardiovascular Imaging Center Weinheim, Hector Foundation, 69469 Weinheim, Germany; 11 Cardiac Imaging, Medneo GmbH Hospital & Health Care, 10117 Berlin, Germany; henning.steen@medneo.com; 12 Cardiology, Angiology and Pneumology, Faculty of Medicine, University of Hamburg, 20148 Hamburg, Germany; 13 Cardiology, Angiology and Pneumology, Medical Faculty, Heidelberg University, 69120 Heidelberg, Germany

**Keywords:** cardiotoxicity, cancer chemotherapy, ventricular strain, cardiac magnetic resonance imaging, ejection fraction

## Abstract

**Background:** Breast and hematological cancer treatments, especially with anthracyclines, have been shown to be associated with an increased risk of cardiotoxicity (CTX). An accurate prediction of cardiotoxicity risk and early detection of myocardial injury may allow for effective cardioprotection to be instituted and tailored to reverse cardiac dysfunction and prevent the discontinuation of essential cancer treatments. **Objectives:** The PRoactive Evaluation of Function to Evade Cardio Toxicity (PREFECT) study sought to evaluate the ability of fast-strain-encoded (F-SENC) cardiac magnetic resonance imaging (CMR) and 2D echocardiography (2D Echo) to stratify patients at risk of CTX prior to initiating cancer treatment, detect early signs of cardiac dysfunction, including subclinical CTX (sub-CTX) and CTX, and monitor for recovery (REC) during cardioprotective therapy. **Methods:** Fifty-nine patients with breast cancer or lymphoma were prospectively monitored for CTX with F-SENC CMR and 2D Echo over at least 1 year for evidence of cardiac dysfunction during anthracycline based chemotherapy. F-SENC CMR also monitored myocardial deformation in 37 left ventricular (LV) segments to obtain a MyoHealth risk score based on both longitudinal and circumferential strain. Sub-CTX and CTX were classified based on pre-specified cardiotoxicity definitions. **Results:** CTX was observed in 9/59 (15%) and sub-CTX in 24/59 (41%) patients undergoing chemotherapy. F-SENC CMR parameters at baseline predicted CTX with a lower LVEF (57 ± 5% vs. 61 ± 5% for all, *p* = 0.05), as well as a lower MyoHealth (70 ± 9 vs. 79 ± 11 for all, *p* = 0.004) and a worse global circumferential strain (GCS) (−18 ± 1 vs. −20 ± 1 for all, *p* < 0.001). Pre-chemotherapy MyoHealth had a higher accuracy in predicting the development of CTX compared to CMR LVEF and 2D Echo LVEF (AUC = 0.85, 0.69, and 0.57, respectively). The 2D Echo parameters on baseline imaging did not stratify CTX risk. F-SENC CMR obtained good or excellent images in 320/322 (99.4%) scans. During cancer treatment, MyoHealth had a high accuracy of detecting sub-CTX or CTX (AUC = 0.950), and the highest log likelihood ratio (indicating a higher probability of detecting CTX) followed by F-SENC GLS and F-SENC GCS. CMR LVEF and CMR LV stroke volume index (LVSVI) also significantly worsened in patients developing CTX during cancer treatment. **Conclusions:** F-SENC CMR provided a reliable and accurate assessment of myocardial function during anthracycline-based chemotherapy, and demonstrated accurate early detection of CTX. In addition, MyoHealth allows for the robust identification of patients at risk for CTX prior to treatment with higher accuracy than LVEF.

## 1. Introduction

There has been an increasing interest in the detection of cardiotoxicity (CTX) at the earliest possible moment to provide safer delivery of cancer treatment. The methods for the detection of cardiovascular dysfunction have evolved substantially over the past decades, but especially with the development of improved and more sensitive cardiac imaging techniques. Recent clinical trials have focused on two-dimensional and three-dimensional echocardiography (Echo) as the most clinically applicable strategy to measure left ventricular ejection fraction (LVEF). Cardiac magnetic resonance imaging (CMR) is widely acknowledged as the gold-standard technique for the assessment of myocardial function with high accuracy and reproducibility [1,2,3,4,5,6].

Although CMR has good inter-study reproducibility and is sensitive for detecting subtle changes in ventricular morphology and function, the lack of widespread availability and physician training with CMR limits its clinical utilization compared to 2D Echo [3,4,5]. Volume-dependent measurements, including CMR and 2D Echo LVEF, are influenced by the volume status of patients, which varies throughout cancer therapy [7,8,9]. This limitation typically hinders the clinical applicability of CMR and 2D LVEF, making it difficult for these measurements to assist clinician decision making. Due to compensatory mechanisms that are operant with variation in loading conditions throughout cancer treatments, the sensitivity of CMR and 2D Echo LVEF for the risk stratification and detection of CTX may be blunted. The major advantage of newer strain algorithms utilized by CMR imaging is direct quantification of myocardial deformation, which is not influenced by volume status. Fast-strain-encoded (F-SENC) allows for volume-independent measurements of myocardial contraction to be accurately quantified in a regional fashion, as well as global measures (e.g., GLS). F-SENC quantifies deformation perpendicular to the image plane on a pixel-based measurement within each voxel providing high spatial resolution and eliminating the potentially confounding impact of changes in heart chamber volume and displacement of the heart wall [10].

The Proactive Evaluation of Function to Evade Cardiotoxicity (PREFECT) trial previously reported the detection of CTX during anthracycline based chemotherapy for cancer utilizing the F-SENC CMR. The present study sought to compare the ability of F-SENC compared to CMR and 2D Echo LVEF for the risk stratification and detection of subclinical and clinical CTX.

## 2. Methods

### 2.1. Study Population

All patients in this prospective cohort study were diagnosed with either breast cancer or lymphoma, were scheduled to receive anthracycline-based chemotherapy, and were treated with curative intent. All patients provided written informed consent at Marien Hospital, Hamburg, Germany between 8/2017 and 7/2019 with local ethics committee (PV5292) approval in compliance with the Declaration of Helsinki. Initial results were published previously [11]. Exclusion criteria included age < 18 or >80 years, inability to give informed consent, pregnancy, previous chemotherapy, contraindications to CMR examination, and renal failure with a glomerular filtration rate < 30 mL/(kg·m^2^). Of 63 consented patients, 3 were excluded due to non-chemotherapy cancer treatment and a 4th missed follow-up visits beyond 1 month. Thus, a total of 59 patients have complete data with over 1 year follow-up that is reported here.

Before initiating chemotherapy, clinical and demographic data, including cardiovascular (CV) risk factors, such as hypertension (HTN), dyslipidemia (Lipid), diabetes mellitus (DM), family history of coronary artery disease (CAD), and smoking status were collected. Medications were recorded throughout the study period including prescribed cardioprotective medications such as angiotensin-converting enzyme inhibitors (ACE-Is), angiotensin II receptor blockers (ARBs), and β-Blockers (BB). Baseline creatinine, BNP (brain natriuretic peptide), and troponin I were obtained. A complete CMR examination with late gadolinium enhancement was performed in all patients before initiating chemotherapy. Baseline evaluation of patient risk was assessed using the HFA-ICOS risk score [12,13], as well as the historical Charlson Comorbidity Index (CCI) [14,15]. The HFA-ICOS risk score was classified according to the following criteria: (1) high risk (3 points)—more than 2 general cardiotoxicity risk factors; presence of severe conditions such as CAD (Coronary Artery Disease), DM, cardiomyopathy (CMP), valvular heart disease (VHD), peripheral artery disease (PAD), atrial fibrillation (AF), or myocardial infarction (MI), CMR LVEF < 54%, age ≥ 65 years; (2) moderate risk (2 points)—one or two general cardiotoxicity risk factors, age between 50 and 64 years, cancer therapy risk factors score of 2; low risk (1 point)—none of the above high or moderate risk criteria are met. Clinical follow-up included medical history with New York Heart Association (NYHA) class assessment, EQ-5D questionnaire, and SF-36 at approximate intervals of 1 month, 3 months, 6 months, and 1 year with standard physical examination including blood testing to assess cardiac biomarkers (troponin I and BNP or NTproBNP) coinciding with F-SENC CMR and 2D Echo imaging.

The attending oncologist managed all cancer treatments according to best practices. Breast cancer (n = 45) patients received 4 cycles of epirubicin and cyclophosphamide in 3-week intervals followed by paclitaxel for 12 weekly cycles (epirubicin, cyclophosphamide, paclitaxel). A few patients were administered dose-dense epirubicin every 2 weeks and cyclophosphamide × 4 followed by 8 cycles of paclitaxel. HER2+ breast cancer patients (n = 10) received trastuzumab. Radiation therapy was administered according to guidelines at the oncology team’s discretion. Non-Hodgkin lymphoma (NHL) patients (n = 11) received cyclophosphamide, doxorubicin, vincristine, and prednisone with the monoclonal antibody rituximab (rituximab–cyclophosphamide, doxorubicin, vincristine and prednisone (R-CHOP)), while Hodgkin lymphoma (HL) (n = 3) patients received anthracycline-based therapy (Table 1).

### 2.2. CMR Examination

CMR scans were performed with a 1.5 Tesla MRI (Achieva, Philips Healthcare, Best, The Netherlands). The CMR protocol for conventional F-SENC and mapping was previously presented [11]. Scan time was approximately 10-to-12 min for follow-up non-contrast CMRs. CMR volumetric data were calculated using cine images from clinical software (cvi42, Circle Cardiovascular Imaging, Inc, Calgary, AB, Canada), as described previously [11]. **F-SENC Acquisition:** F-SENC images were analyzed with MyoStrain^®^ software version 5.2.4 (Myocardial Solutions, Inc, Morrisville, NC, USA). Short-axis images were used to quantify longitudinal strain (LS) while long-axis images were used to quantify circumferential strain (CS). GLS was the mean midmyocardial peak longitudinal strain for all short-axis left ventricular (LV) segments (n = 16), while global CS (GCS) was the mean of all long-axis LV segments (n = 21), as described previously [16]. Peak end-systolic midmyocardial LS or CS in any segment ≤ −17% was considered normal [17,18]. The % of normal segments (e.g., MyoHealth) is the # of normal LS and CS LV segments (≤−17%) divided by the total number of segments analyzed (max 37 segments) [16]. LS and CS were measured and are reported as negative numbers to reflect myocardial contraction/deformation. **Follow-Up CMR Examinations:** Patients received F-SENC CMR follow-up scans at regular time intervals based on cancer treatment [e.g., after 150 mg/m^2^ doxorubicin (or 270 mg/m^2^ epirubicin), upon anthracycline completion, and 6 and 12 months after cancer treatment initiation. Additional clinical examinations and F-SENC CMR scans were acquired based on patient symptoms or clinical status [11].

### 2.3. Echocardiography Measurements

Echocardiograms (2D Echo) were obtained at coincident time intervals to CMR, and included a standard comprehensive assessment of LVEF, left ventricular end-diastolic volume indexed to the body surface area, mL/m^2^ (LVEDVi), left ventricular end-systolic volume index (LVESVi), and left ventricular stroke volume index (LVSVi). The 2D Echo was performed with a Philips Affiniti 50 (Best, The Netherlands). Two-dimensional Echo LVEF was measured by Simpson’s method from biplane measurements from the 2- and 4-chamber views according to current recommendations. All 2D Echo images and measurements were reviewed twice by expert reviewers who were not aware of the clinical events.

### 2.4. Definitions of Cardiotoxicity (CTX)

The development of symptomatic heart failure (HF), a significant reduction in EF, or biomarker indicators of myocardial injury were used to classify patients with subclinical cardiotoxicity (Sub-CTX) and clinical cardiotoxicity (CTX) consistent with the ASE/EACVI Expert Consensus Position Paper and the ESC Position Paper for CV toxicity, as previously summarized [11,19,20]. Importantly, MyoHealth was not used for the differentiation between absent, subclinical, and manifest CTX. Sub-CTX included asymptomatic patients with a decrease in LVEF ≥ 10% with absolute value ≥ 53%, decrease in GLS more than 15% from baseline, or abnormal cardiac biomarkers (troponin I, BNP, or NTproBNP). Clinical CTX included an absolute reduction in LVEF ≥ 10% from baseline to below 53% combined with HF symptoms or abnormal cardiac biomarkers (troponin, BNP, or NTproBNP). These parameters of cardiotoxicity were the prevailing prespecified definitions at the time of this trial (2017–2019), and were since updated by ESMO and ESC CardioOncology guideline statements more recently [21,22]. Cardiac recovery (REC) during cardioprotective therapy was classified as patients exhibiting sub-CTX or clinical CTX who experienced improvement of clinical symptoms, LVEF, GLS, and/or cardiac biomarkers, as defined by the treating physicians. Patients classified as Sub-CTX or CTX were monitored and managed using cardioprotective medications at the discretion of the treating physicians. Patients with HTN at baseline were commonly on anti-hypertensive medications pre-chemotherapy, which were adjusted based on patient response.

### 2.5. Statistical Analysis

Data are presented as the mean ± standard deviation (SD) for normally distributed continuous variables, or as counts and percentage for categorical variables. Comparing 3+ groups of continuous variables was tested using an ANOVA with the Scheffé test for post hoc analysis. The Mann–Whitney test compared ordinal variables, while nominal variables were compared using the Fisher test. Patient differences based on CTX status were compared using the Kruskal–Wallis test, while the CTX groups were compared using Conover post hoc analysis. Box and Whisker’s plots were created to graphically represent the mean, median, interquartile range (IQR), and extent of values within 1.5 times the IQR, where values outside that range are highlighted as outliers.

Receiver operating characteristic (ROC) analysis was performed to determine the optimal cutoff values for sensitivity and specificity in detecting CTX for each imaging parameter. Comparison of the areas under the curve (AUC) of paired data ROC curves was performed using the DeLong method [23]. Logistic regression analysis used the maximum likelihood estimation (MLE) method to model the relationship between the log odds of a Sub-CTX or CTX event for each independent imaging parameter, including MyoHealth, F-SENC GLS, F-SENC GCS, CMR LVEF, CMR LVSVi, 2D Echo LVEF, and 2D Echo LVSVi. The log likelihood ratio test (LLR) compared a full model with all predictors and a reduced model missing each imaging parameter to test the significance of each excluded imaging parameter where the LLR statistic = −2 * (log likelihood of reduced model; log-likelihood of full model). A Chi-square distribution was used to evaluate statistical significance of the LLR statistic. Statistical significance was defined by *p* ≤ 0.05.

## 3. Results

A total of 59 patients were prospectively enrolled, included in the analysis, and followed sequentially for at least one year after enrollment. The demographics are displayed in Table 1. Patients with no CTX throughout follow-up (n = 26), sub-CTX during follow-up (n = 24), and CTX during follow-up (n = 9) were not different for baseline characteristics, with the exception of age and CCI, where the CTX group was older (66 ± 10 y vs. 54 ± 14 y for all, *p* = 0.02) and had a higher CCI (3.8 ± 1.7 vs. 1.8 ± 1.9 for all, *p* = 0.002). The baseline vitals did not differ between groups. The CMR parameters at baseline in the CTX group were notable for a lower LVEF (57 ± 5% vs. 61 ± 5% for all, *p* = 0.05), as well as a lower MyoHealth (70 ± 9 vs. 79 ± 11 for all, *p* = 0.004) and a worse F-SENC GCS (−18 ± 1 vs. −20 ± 1 for All, *p* < 0.001). Additionally, the septal wall thickness was increased in the CTX group (10 ± 3 mm vs. 8 ± 2 mm for all, *p* = 0.014). Blank *p*-values in Table 1 and Table 2 represent non-significant comparisons, where *p* > 0.05.

**Figure 1:**
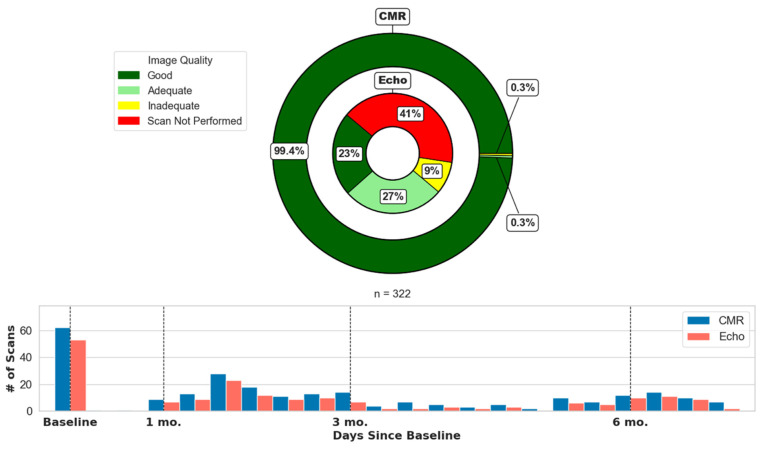
Image quality and timing for CMR and 2D Echo exams.

The data obtained during follow-up CMR and 2D Echo exams are provided in Table 2 and Figure 1. F-SENC CMR images exhibited good quality on at least 5/6 slices in 320/322 (99.4%) scans, with excellent image quality on all 6 slices in 314/322 (97.5%). The 2D Echo image quality was good in at least 2/3 long-axis slices to obtain LVEF calculations in 73/322 (23%) exams, with excellent 2D Echo image quality in 3/3 long-axis slices in 46/322 (14%) exams. The 2D Echo LVEF was calculated despite poor image quality in two or three long-axis images in 110/322 (34%) exams, while 2D Echo LVEF was unable to be calculated in 6/322 (2%) exams. Two-dimensional Echo was not performed in 133/322 (41%) of scheduled scans due to radiation along the chest wall for breast cancer or lymphoma in 34/133 (26%), operation along the chest wall for breast cancer or lymphoma in 2/133 (2%), prior echocardiograms with poor image quality in 55/133 (41%), or scheduling conflicts/patient choice in 42/133 (32%) of cases.

Over the course of the PREFECT trial, the vitals did change depending on CTX status (Table 2), all analyzed by ANOVA comparisons. The heart rate was higher in the Sub-CTX and CTX compared to the No CTX group (81 ± 13 and 76 ± 8 vs. 71 ± 11 bpm, respectively, *p* < 0.001). The systolic blood pressure (SBP) was higher in the CTX group compared to all the other groups (130 ± 20 mmHg vs. 120 ± 17 mmHg, *p* = 0.019). The diastolic blood pressure (DBP) was higher in the Sub-CTX and CTX groups compared to the No CTX group (79 ± 9 mmHg and 80 ± 9 vs. 72 ± 10 mmHg, *p* < 0.001). Every F-SENC CMR determined parameter was able to detect significant differences between the No CTX, Sub-CTX, and CTX groups, as well as those patients who recovered (REC) from CTX (all highly significant at *p* < 0.001, except *p* = 0.03 for LVEDVi).

**Figure 2:**
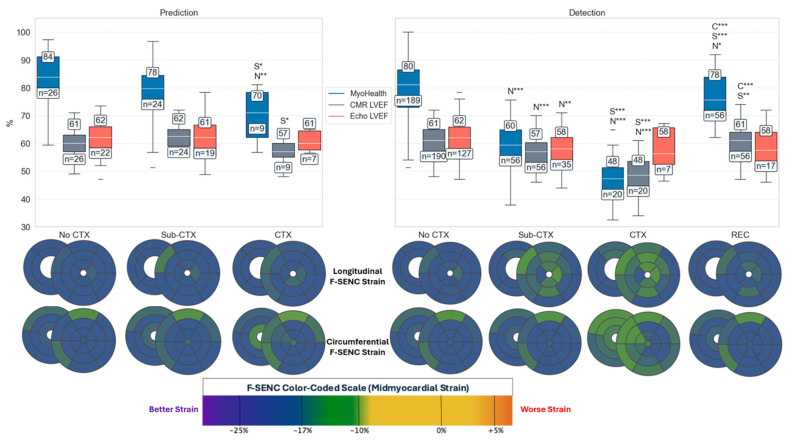
Box and Whisker’s plots of baseline and follow-up MyoHealth, CMR LVEF, and 2D Echo LVEF for the risk prediction and detection of CTX with regional F-SENC polar plots of average peak end-systolic segmental values by CTX status. Significance comparison: N = No CTX, S = Sub-CTX, C = CTX. * *p* < 0.05; ** *p* < 0.01; *** *p* < 0.001.

Figure 2 summarizes the cardiotoxicity group delineation for MyoHealth, CMR LVEF, and 2D Echo LVEF measurements at baseline, pre-chemotherapy assessment for the prediction of cardiotoxicity (left side panel), and the follow-up data for the detection (right side panel) of cardiotoxicity and recovery (REC) by Kruskal–Wallis test comparisons. Polar plots of segmental LS (top) and CS (bottom) F-SENC show end-systolic midmyocardial strain, where blue represents peak contraction ≤ −17%, green represents peak contraction between −10 and −17%, and yellow represents peak contraction > −10%. Patients who developed CTX had a reduced F-SENC septal CS at baseline compared to the No CTX and Sub-CTX groups.

A reduced MyoHealth (% LS and CS segments ≤ −17) at baseline was significantly lower in CTX (70 ± 9) when compared to the Sub-CTX (78 ± 11, *p* < 0.05) and No CTX groups (84 ± 10, *p* < 0.01), while the CMR LVEF at baseline was only able to predict subsequent CTX (57 ± 5%) when compared to the Sub-CTX group (62 ± 5%, *p* < 0.05). Baseline 2D Echo LVEF was not able to accurately predict CTX. During cancer treatment, MyoHealth detected Sub-CTX (60 ± 9, *p* < 0.001), CTX (48 ± 9, *p* < 0.001), and REC (78 ± 8, *p* < 0.05) in a highly significant manner when compared to the No CTX (80 ± 10) group. CMR LVEF detected the Sub-CTX (57 ± 6, *p* < 0.001) and CTX (48 ± 7%, *p* < 0.001) groups compared to the No CTX (61 ± 5%) group, and REC (61 ± 6%) was improved and highly significant when compared to the Sub-CTX (*p* < 0.01) and CTX (*p* < 0.001) groups. Two-dimensional Echo LVEF did not predict any CTX status and only detected Sub-CTX (58 ± 7%) when compared to the No CTX (62 ± 6%, *p* < 0.01) group.

**Figure 3:**
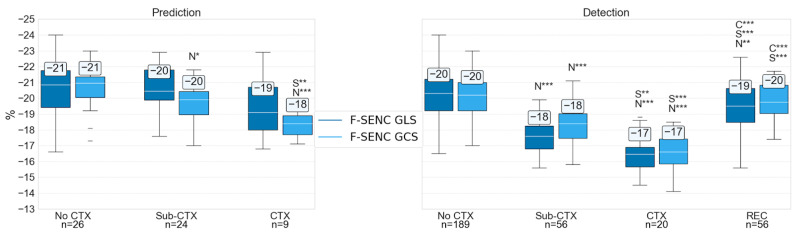
F-SENC GLS and GCS for risk prediction, detection, and monitoring of CTX. Significance comparison: N = No CTX, S = Sub-CTX, C = CTX. * *p* < 0.05; ** *p* < 0.01; *** *p* < 0.001.

Figure 3 illustrates the detailed myocardial strain assessments using F-SENC (GLS and GCS). When compared to No CTX (−20 ± 1), F-SENC demonstrated a highly significant worsening in GLS and GCS in both Sub-CTX (−18 ± 1, *p* < 0.001) and CTX (−17 ± 1, *p* < 0.001) groups, while also revealing a highly significant improvement in the REC group (−20 ± 1, *p* < 0.001).

**Figure 4:**
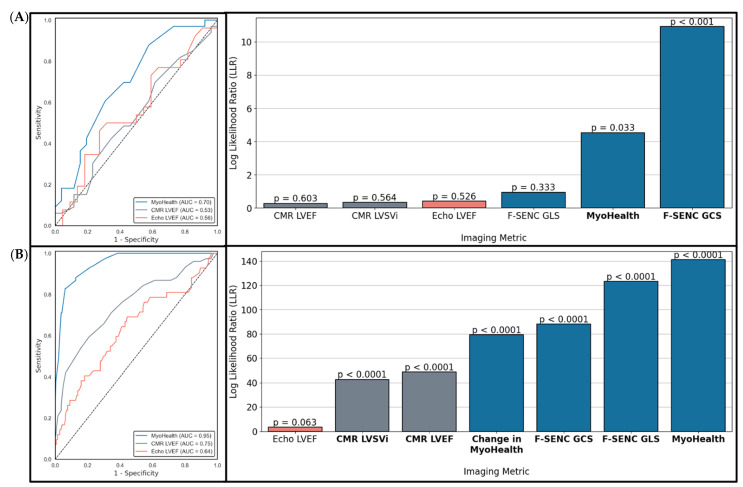
(**A**,**B**) ROC and LLR of baseline imaging metrics for (**A**) the risk prediction of Sub-CTX or CTX, and follow-up exams for (**B**) the detection of Sub-CTX or CTX.

Figure 4 illustrates the logistic regression data for risk stratification and the detection of cardiotoxicity. All F-SENC CMR observations were statistically significant, while 2D Echo LVEF had limited statistical significance (only the comparison between Sub-CTX and No CTX). As shown in the ROC and LLR analyses in Figure 4A,B, MyoHealth and F-SENC GCS were the only two statistically significant imaging parameters during baseline, pre-chemotherapy LLR analyses for risk stratification of incident CTX, while MyoHealth had a higher AUC (0.70) compared to CMR LVEF (0.53) and 2D Echo LVEF (0.56). All F-SENC parameters showed statistical significance for LLR analyses with MyoHealth having the highest LLR (indicating a higher probability of detecting CTX) followed by F-SENC GLS and F-SENC GCS. CMR LVEF and CMR LVSVI also significantly worsened in patients developing CTX during cancer treatment, while 2D Echo LVEF did not. These results were enhanced by the ROC analyses where the AUC for MyoHealth (0.95) was much higher than that for CMR LVEF (0.75), and 2D Echo LVEF (0.64).

**Figure 5:**
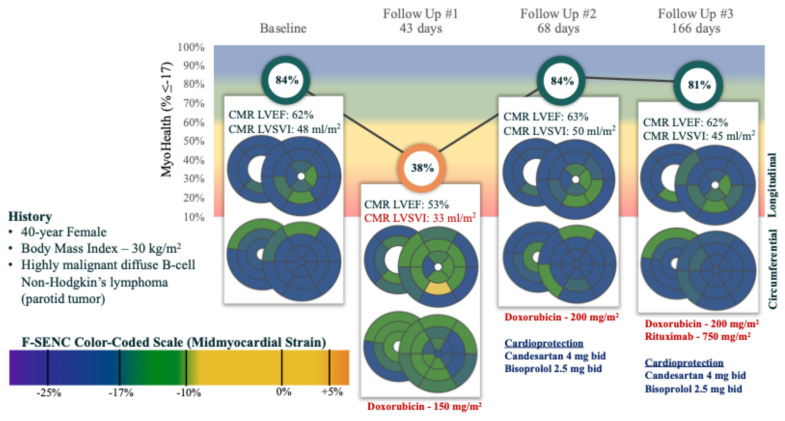
Representative case illustrating the sensitivity of MyoHealth for detecting Sub-CTX and monitoring REC during anthracycline-based cancer treatment. Legend: this patient had evidence of cardiotoxicity with intermediate dosing of anthracycline that was very evident by a substantial reduction in MyoHealth score, while the CMR LVEF did not meet criteria for cardiotoxicity. Once cardioprotective therapy was instituted, the MyoHealth score was normalized and the patient completed the planned anthracycline treatment maintaining normal cardiac function despite ongoing cardiotoxic therapy.

**Figure 6:**
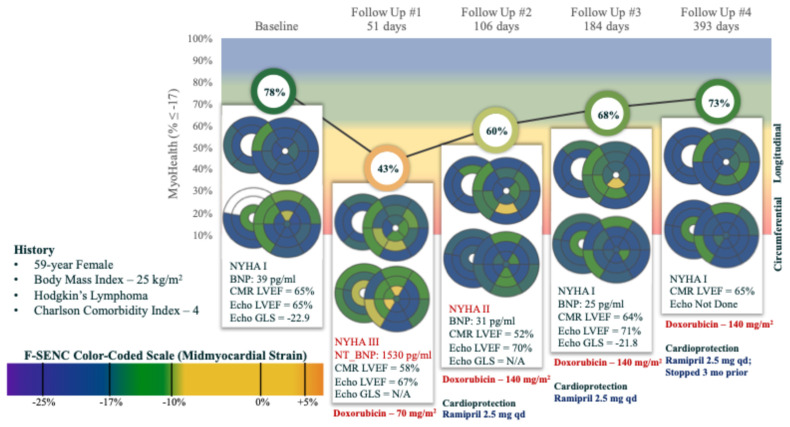
Representative case illustrating the responsiveness of MyoHealth for detecting Sub-CTX and REC with cardioprotection during ongoing anthracycline treatment. Legend: this patient had a normal LVEF by Echo throughout chemotherapy although cardiotoxicity was confirmed by CMR LVEF, symptoms, and biomarkers. The MyoHealth score was normal at baseline but was markedly reduced after one dose of anthracycline while the CMR LVEF remained normal. Once cardioprotective therapy was instituted and the patient continued anthracycline chemotherapy, the MyoHealth score improved and ultimately returned to normal values. The MyoHealth score was much more sensitive to ongoing damage and subsequent improvement with cardioprotection than LVEF by either CMR or Echo.

Figure 5 and Figure 6 detail the successive CMR LVEF, Echo LVEF, cardiac biomarkers and MyoHealth scores in two individual patients, as they underwent anthracycline based chemotherapy for cancer. Figure 5 highlights the sensitivity of changes in MyoHealth in contrast to CMR LVEF for the detection of cardiotoxicity and responsiveness to enhanced cardioprotective therapy. Figure 6 is a demonstration of the sensitivity of MyoHealth to early detection when compared to LVEF and the insensitivity of Echo LVEF to evidence ongoing toxicity or recovery over the course of anthracycline chemotherapy.

Additional analyses can be found in the Supplementary Materials.

## 4. Discussion

In the PREFECT study, serial F-SENC CMR and 2D Echo were prospectively performed in patients undergoing anthracycline-based chemotherapy during their cancer treatment. The traditional availability limitations of CMR are mitigated by F-SENC, which enables a 10–12-min non-contrast exam [11]. F-SENC CMR was reliable and accurate in detecting cardiotoxicity, with >99% of all F-SENC CMR images providing LVEF, LV volumes, and myocardial strain (Figure 1). The baseline assessment of LVEF by 2D Echo was unable to identify patients at risk for subsequent cardiotoxicity, while multiple F-SENC CMR parameters (LVEF, septal wall thickness, MyoHealth, and GCS) were able to accurately stratify those at a risk with a high degree of significance (Table 1).

F-SENC CMR provides an alternative to 2D Echo in patients exposed to radiation or surgery involving the chest wall on the left side, which may hinder transducer manipulation and cause discomfort and/or poor image quality. This is especially relevant in patients with left-sided breast cancer, who require cardiovascular safety monitoring with myocardial imaging. F-SENC CMR offers an obtainable and accurate cardiac assessment while 2D Echo imaging may not be effective for imaging in a sizable percentage of the breast cancer and lymphoma population, especially in obese patients, those with prior left chest surgery, and those with breast reconstruction due to poor or limited echogenic windows [24,25,26,27]. In this population, for those that require comparison of measurements from serial exams to detect changes consistent with cardiotoxicity from cancer treatment, F-SENC CMR may represent a preferred imaging modality [3].

This prospective study emphasizes the advantages of F-SENC CMR in stratifying the risk of CTX and detecting early CTX with accurate measurements of LVEF and myocardial strain (using the F-SENC method, which includes GLS and GCS, which are incorporated into the MyoHealth score). The baseline data in Table 1 highlight this observation, indicating that a lower LVEF by CMR was able to significantly stratify patients at a high risk of developing CTX, while a lower MyoHealth was an even more significant predictor of subsequent cardiotoxicity (*p* = 0.004). The improved prediction by F-SENC GCS is in line with previous data, suggesting circumferential dysfunction indicates an underlying cardiomyopathy due to pre-existing cardiac conditions and increases morbidity and mortality risk [28]. Intramyocardial circumferential dysfunction in asymptomatic patients with a normal LVEF and no history of structural heart disease identifies a higher risk of adverse events, as demonstrated in the Multi-Ethnic Study of Atherosclerosis [29]. In addition, circumferential F-SENC strain, especially along the septum, has been shown to delineate dysfunction from subclinical CV diseases with a higher accuracy than longitudinal strain [16]. Considering that potentially cardiotoxic cancer treatments classically affect the entire heart, regions exhibiting damage from pre-existing conditions (primarily septal circumferential segments, as shown in Figure 2) are subject to even more injury during cancer treatments which can overwhelm the heart’s natural compensatory mechanisms, resulting in global myocardial dysfunction.

The reliable identification of regional myocardial dysfunction before global myocardial damage allows for tailored cardioprotective therapy to be instituted during cancer treatment. This process can help ensure that cancer treatment continues uninterrupted while proactively preventing cardiac damage or enhancing myocardial recovery from transient injury. Figure 2 and Figure 3, as well as Table 2 delineate the ability of F-SENC CMR (using GLS and GCS) to document the recovery of cardiac function to baseline values while chemotherapy continues to optimally treat the underlying cancer. Previous studies have attempted to demonstrate this principle of enhanced cardioprotection while continuing potentially cardiotoxic cancer therapy, but the magnitude of change of any imaging parameter was not enough to be clinically meaningful [30,31,32]. The accuracy of F-SENC CMR, which quantifies myocardial deformation without incorporating volume-dependent algorithms of displacement, allows for clinical decisions to be adjusted based on a reliable change in serial measurements, indicating improvement or worsening. By having actionable parameters to respond to during chemotherapy, cardioprotection can be optimized, providing the ability to continue cancer treatments uninterrupted.

Serial measurements during the chemotherapy of F-SENC CMR highlighted significant differences between cardiotoxicity groups for the risk stratification and early detection of cardiotoxicity. When examining the likelihood that a baseline (pre-chemotherapy) imaging parameter abnormality would risk stratify subsequent CTX or Sub-CTX, only MyoHealth and F-SENC GCS were significantly associated with the incidence of cardiotoxicity. During chemotherapy, F-SENC CMR parameters were able to detect Sub-CTX, CTX or REC, and MyoHealth was the most highly significant.

Additional analyses can be found in the Supplementary Materials, including more detailed MRI techniques, extensive dosing description of chemotherapy utilized during the treatment phase of this study, and exploratory myocardial contraction timing analyses as indicators of cardiotoxicity.

## 5. Study Limitations

The PREFECT trial was a prospective study evaluating serial scans of F-SENC CMR and 2D Echo for monitoring heart function during anthracycline-based chemotherapy to allow for the risk stratification and early detection of cardiotoxicity. This was a single-center non-randomized study in a limited patient population, but both imaging tools were obtained in a coincident manner in the majority of patients. It is recognized that a larger study would have been preferable, but the detailed analysis of this study allowed us to demonstrate important and significant differences in monitoring techniques despite the relatively small patient population. Another limitation of sophisticated CMR imaging is availability in a broad population of patients undergoing cancer treatment. While F-SENC CMR is not available to all patients undergoing cancer treatment at this point, several characteristics of this technique allow for wider utilization. Automated regional strain computation is available within minutes of the scan being completed in a readily interpretable report, and is therefore an actionable tool for clinicians. The scan protocol also has a much shorter time requirement (10–15 min) than conventional CMR exams, as well as no need for contrast injection and LGE analysis.

## 6. Conclusions

F-SENC CMR quantified myocardial function during anthracycline-based chemotherapy, and demonstrated high clinical utility for the risk stratification, early detection, and identification of the recovery of cardiac function in patients receiving and completing aggressive cancer treatment. MyoHealth and F-SENC GCS allow for the robust identification of cancer patients at risk for cardiotoxicity before initiating chemotherapy. Several F-SENC CMR indicators of cardiac dysfunction during cancer treatment enable cardioprotection to be instituted while monitoring F-SENC CMR parameters, particularly the MyoHealth score, enabling tailored cardioprotective therapy to enhance the recovery of myocardial injury. Further studies are now warranted to detect the most effective strategy for the risk stratification and early detection of CTX, and the documentation of the effectiveness of cardioprotective treatment, which may be life-saving in patients with severe CTX.

## Figures and Tables

**Table 1:** Demographics and baseline measurements for the study population.

	All Patients (n = 59)	No CTX (n = 26)	Sub-CTX (n = 24)	CTX (n = 9)	*p*-Value
**Demographics and Clinical History**					
Age, y	54 ± 14	52 ± 13	51 ± 16	66 ± 9.6	**0.02**
Female, n (%)	50 (85)	24 (92)	20 (83)	6 (67)	
BMI, kg/m^2^	26 ± 5	25 ± 5	27 ± 6	25 ± 4	
HTN, n (%)	21 (36)	8 (31)	9 (38)	4 (44)	
Lipid, n (%)	12 (20)	4 (15)	5 (21)	3 (33)	
DM, n (%)	4 (7)	2 (8)	1 (4)	1 (11)	
TOB, n (%)	19 (32)	8 (31)	7 (29)	4 (44)	
HER2−, n (%)	35 (59)	16 (62)	16 (67)	3 (33)	
HER2+, n (%)	10 (17)	7 (27)	2 (8)	1 (11)	
NHL, n (%)	11 (19)	3 (12)	5 (21)	3 (33)	
HL, n (%)	3 (5)	0 (0)	1 (4)	2 (22)	
CCI	1.8 ± 1.9	1.4 ± 1.6	1.6 ± 1.8	3.8 ± 1.7	**0.002**
HFA-ICOS Score	1.5 ± 0.8	1.4 ± 0.7	1.5 ± 0.7	2.0 ± 1.0	
**Baseline Vitals**					
Heart rate, bpm	72 ± 11	70 ± 12	75 ± 10	72 ± 11	
SBP, mmHg	124 ± 18	119 ± 14	127 ± 21	131 ± 19	
DBP, mmHg	75 ± 11	73 ± 10	77 ± 11	77 ± 13	
**Baseline F-SENC CMR**					
LVEF, %	61 ± 5	61 ± 5	62 ± 5	57 ± 5	**0.05**
LVEDVi, mL/m^2^	75 ± 13	75 ± 12	74 ± 13	76 ± 11	
LVESVi, mL/m^2^	29 ± 7	30 ± 8	28 ± 7	33 ± 7	
LVSVi, mL/m^2^	45 ± 8	45 ± 7	46 ± 9	44 ± 6	
Septal wall thickness, mm	8 ± 2	8 ± 2	9 ± 2	10 ± 3	**0.01**
LGE Present, n (%)	19 (32)	9 (35)	7 (29)	3 (33)	
MyoHealth (% ≤ −17)	79 ± 11	84 ± 10	78 ± 11	70 ± 9	**0.004**
F-SENC GLS, %	−20 ± 2	−21 ± 2	−20 ± 2	−19 ± 2	
F-SENC GCS, %	−20 ± 1	−21 ± 1	−20 ± 1	−18 ± 1	**<0.001**
**Baseline 2D Echo**					
LVEF, %	62 ± 7	62 ± 7	61 ± 8	61 ± 4	
LVEDVi, mL/m^2^	45 ± 13	42 ± 10	47 ± 17	48 ± 8	
LVESVi, mL/m^2^	18 ± 8	16 ± 5	19 ± 10	20 ± 6	
LVSVi, mL/m^2^	27 ± 8	26 ± 6	28 ± 10	28 ± 6	

BMI—body mass index; HT—arterial hypertension; Lipid—hyperlipidemia; DM—diabetes mellitus; TOB—current/prior smoker; HER2−—HER2-negative breast cancer; HER2+—HER2-positive breast cancer; NHL—non-Hodgkin’s lymphoma; HL—Hodgkin’s lymphoma; CCI—Charlson comorbidity index; SBP—systolic blood pressure; DBP—diastolic blood pressure; LV—left ventricular; EF—ejection fraction; EDVi—end-diastolic volume index; ESVi—end-systolic volume index; SVi—stroke volume index; LGE—late gadolinium enhancement; GLS—global longitudinal strain; GCS—global circumferential strain.

**Table 2:** Vitals and imaging measurements throughout cancer treatment.

	All Scans (n = 322)	No CTX (n = 190)	Sub-CTX (n = 56)	CTX (n = 20)	REC (n = 56)	*p*-Value
**Follow-Up Visit Vitals**						
Heart rate, bpm	73 ± 12	71 ± 11	81 ± 13	76 ± 8	73 ± 9	**<0.001**
SBP, mmHg	120 ± 17	120 ± 18	120 ± 14	130 ± 20	120 ± 17	**0.02**
DBP, mmHg	74 ± 10	72 ± 10	79 ± 9	80 ± 9	72 ± 10	**<0.001**
**F-SENC CMR**						
LVEF, %	59 ± 6	61 ± 5	57 ± 6	48 ± 7	61 ± 6	**<0.001**
LVEDVi, mL/m^2^	76 ± 12	77 ± 12	73 ± 15	76 ± 10	73 ± 13	**0.03**
LVESVi, mL/m^2^	31 ± 8	30 ± 7	31 ± 9	40 ± 10	29 ± 9	**<0.001**
LVSVi, mL/m^2^	45 ± 8	47 ± 7	41 ± 8	36 ± 5	44 ± 7	**<0.001**
Septal wall thickness, mm	8 ± 2	7 ± 2	8 ± 2	10 ± 2	8 ± 2	**<0.001**
MyoHealth (% ≤ −17), %	74 ± 14	80 ± 10	60 ± 9	48 ± 9	78 ± 8	**<0.001**
F-SENC GLS, %	−19 ± 2	−20 ± 1	−18 ± 1	−17 ± 1	−19 ± 2	**<0.001**
F-SENC GCS, %	−20 ± 2	−20 ± 1	−18 ± 1	−17 ± 1	−20 ± 1	**<0.001**
**2D Echo**						
LVEF, %	61 ± 7	62 ± 6	58 ± 7	58 ± 8	58 ± 7	**0.003**
LVEDVi, mL/m^2^	45 ± 11	46 ± 11	45 ± 12	43 ± 11	41 ± 9	
LVESVi, mL/m^2^	17 ± 6	17 ± 6	18 ± 6	19 ± 8	15 ± 6	
LVSVi, mL/m^2^	28 ± 8	28 ± 8	27 ± 8	26 ± 4	26 ± 6	

SBP—systolic blood pressure; DBP—diastolic blood pressure; LV—left ventricular; EF—ejection fraction; EDVi—end-diastolic volume index; ESVi—end-systolic volume index; SVi—stroke volume index; GLS—global longitudinal strain; GCS—global circumferential strain.

## Data Availability

The study data are available upon reasonable request from the corresponding author.

## Supplementary Materials

The following supporting information can be downloaded at: https://www.mdpi.com/article/10.3390/diagnostics15151948/s1, Figure S1: Histogram plot of CMR LVEF (A), 2D Echo LVEF (B), and F-SENC MyoHealth (C) with the counts listing the number of exams for the detection of each CTX classification.; Figure S2: F-SENC timing metrics (SI and SD-TPS) for the prediction of CTX, and the detection of CTX and REC; Figure S3: ROC curves of F-SENC timing metrics (SI and SD-TPS) for CTX prediction (A), and CTX detection (B). Table S1: Cancer treatments and cardioprotective medications according to CTX status [33,34,35,36].
