# Supplementary material for: Myocardial Strain Measurements Obtained with Fast-Strain-Encoded Cardiac Magnetic Resonance for the Risk Prediction and Early Detection of Chemotherapy-Related Cardiotoxicity Compared to Left Ventricular Ejection Fraction
Source: Diagnostics (Basel). 2025 Aug 3;15(15):1948. doi: 10.3390/diagnostics15151948 (PMC12346548; doi:10.3390/diagnostics15151948)
Supplement: Supplementary file 1 [file diagnostics-15-01948-s001.zip › diagnostics-3753300-supplementary.pdf]

Supplemental Files

Detailed CMR methods utilized in all studies:

T1 maps were acquired using a standard Modified Look-Locker Inversion Recovery 5s(3s)3s T1-native sequence in standard midventricular short-axis views. T2 Maps were acquired using an mGRASE sequence with 9 echos. Lastly, 6 F-SENC acquisitions were performed by comparing low- and high-tuning frequency modulation image sequences encoded from +5% to -30% that represent static and maximally contracting states (3 short axes: basal, mid, and apical; 3 long axes: 4-chamber, 2-chamber, and 3-chamber). Single heartbeat, F-SENC pulse sequence with single-shot spiral readouts utilized the following imaging parameters as previously described: field-of-view=256×256 mm^2, slice thickness=10 mm, voxel size=4×4×10 mm^3, reconstructed resolution=1×1× 10 mm3, single-shot spiral readout with acquisition time TA=10ms, flip angle=30°, effective echo time (TE)=0.7ms, repetition time (TR)=12ms, temporal resolution=36ms, typical number of acquired heart phases=22, spectrally selective fat suppression (SPIR), total acquisition time per slice <1s. [1]

F-SENC CMR Timing Metrics: F-SENC timing metrics were obtained by automatically contouring all frames for each image sequence to generate midmyocardial strain curves for each segment and integrating all image sequences into a composite model to compare various timing metrics regionally and globally. The similarity of strain curves from every region (longitudinal and circumferential) of the heart were compared based on a Spearman correlation of all strain curves to calculate a global synchronicity index (**SI**). A **SI** close to 1.0 describes highly synchronized contraction throughout the heart. The time from the ECG gated R-wave to peak end-systolic strain (**TPS**) was taken for each longitudinal and circumferential segment so the variability in time to peak strain could be calculated as the standard deviation (**SD**) of time to peak strain (**SD-TPS**) where **SD-TPS** < 50 msec describe highly synchronized contraction. [1] [2] [3] [4]

SUPPLEMENTAL TABLES & FIGURES

Supplemental Table S1. Cancer Treatments & Cardioprotective Medications by CTX Status

	All Scans (n = 322)	No CTX (n = 190)	Sub-CTX (n = 56)	CTX (n = 20)	REC (n = 56)
**Cancer Treatment**					
Doxorubicin	217 ± 81 (19)	175 ± 65 (7)	215 ± 84 (32)	198 ± 90 (65)	263 ± 63 (32)
Epirubicin	340 ± 38 (61)	334 ± 41 (59)	336 ± 40 (68)	347 ± 34 (35)	360 ± 0 (68)
Taxane	300 ± 430 (22)	250 ± 400 (25)	310 ± 430 (34)	120 ± 260 (15)	530 ± 460 (29)
Trastuzumab	0.6 ± 2.6 (7)	0.7 ± 2.9 (11)	0.2 ± 0.6 (7)	0 ± 0 (0)	0.8 ± 3.3 (7)
Pertuzumab	0.4 ± 2.2 (4)	0.8 ± 3.1 (8)	0.1 ± 0.4 (2)	0 ± 0 (0)	0.5 ± 2.4 (5)
Rituximab	136 ± 571 (4)	26 ± 150 (2)	80 ± 340 (5)	394 ± 922 (15)	502 ± 1109 (9)
Carboplatin	18 ± 204 (8)	41 ± 320 (9)	1 ± 4 (11)	0 ± 0 (0)	3 ± 5 (12)
Radiation	1.3 ± 5.2 (5)	0.8 ± 4.4 (2)	0.7 ± 3.7 (5)	1.1 ± 4.9 (5)	3.5 ± 7.8 (14)
**Cardioprotective Medications**					
Oral anticoagulants	14 (4)	7 (4)	3 (5)	2 (10)	2 (4)
Statins	33 (10)	6 (3)	10 (18)	6 (30)	11 (20)
Calcium antagonists	12 (4)	7 (4)	1 (2)	0 (0)	4 (7)
ß-blocker	110 (34)	34 (18)	24 (43)	12 (60)	40 (71)
ACE-I	37 (12)	6 (3)	9 (16)	10 (50)	12 (21)
ARB	85 (26)	30 (16)	15 (27)	2 (10)	38 (68)
**Any Diuretic**	50 (16)	21 (11)	12 (21)	9 (45)	8 (14)
K-Sparing Diuretic	11 (3)	2 (1)	4 (7)	3 (15)	2 (4)
Thiazide Diuretic	27 (8)	14 (7)	6 (11)	4 (20)	3 (5)
Loop Diuretic	12 (4)	5 (3)	2 (4)	2 (10)	3 (5)

Anthracycline, Taxane, and Rituximab are listed as cumulative dosages (mg/m^2) followed by (%) of scans in which patient was receiving indicated cancer treatment. Trastuzumab, Pertuzumab, Carboplatin, and Radiation treatments were listed as # cycles followed by (%) of scans patient was receiving indicated treatment. Cardioprotective Medications are presented as total number and percent of scans, n (%), in which patients in each CTX classification were taking individual cardio-active medication

Supplemental Figure S1 (A to C). Histogram Plot of CMR LVEF (A), 2D Echo LVEF (B) and F-SENC MyoHealth (C) with the counts listing the number of exams for detection of each CTX classification.

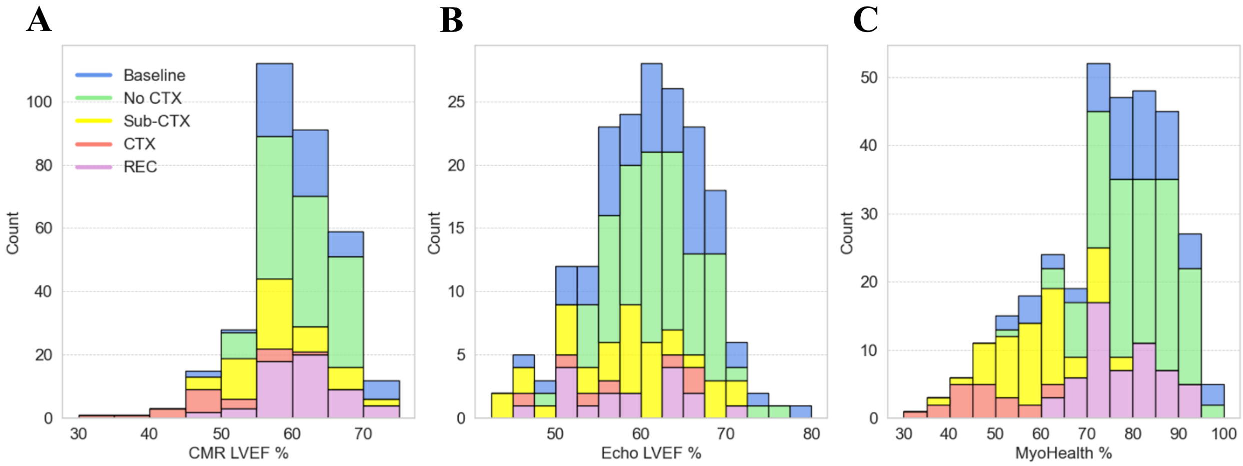

The number of observations throughout cancer treatment delineated by CTX status are shown in supplemental Figures S1A to C to highlight the ability to each individual parameter to detect CTX. F-SENC MyoHealth showed the best delineation of CTX where most values below 65% were associated with Sub-CTX or CTX and most values above 70% were pre-chemotherapy baseline, no cardiotoxicity (No CTX), or recovery (REC) to baseline function. CMR LVEF observed a high percentage of CTX below 50% while 2D Echo LVEF was discordant from the CMR parameters where several exams where 2D Echo LVEF were normal observed poor MyoHealth and CMR LVEF.

Supplemental Figure S2 (A and B). F-SENC Timing Metrics (SI & SD-TPS) for Prediction of CTX, and Detection of CTX & REC

Baseline (Prediction) Follow-Ups (Detection)

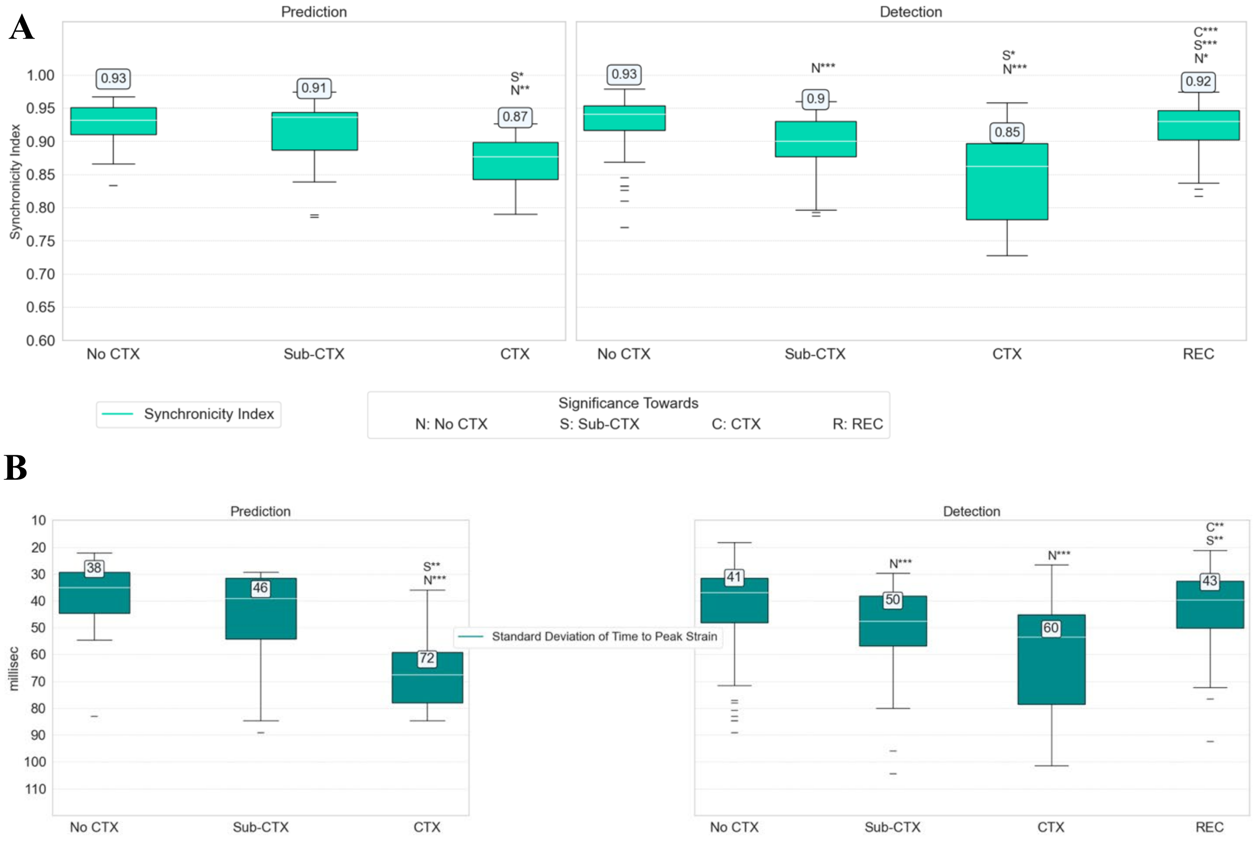

Significance Comparison: N = No CTX, S = Sub-CTX, C = CTX

*p<0.05; **p<0.01; ***p<0.001

Synchronicity index (SI) and standard deviation of time to peak strain (SD-TPS) are both indicators of mechanical coordination and quantify the similarity in shape and/or timing of regional or segmental strain curves.

Synchronicity index (SI) is calculated from a spearman correlation of all longitudinal and circumferential regional strain curves where a SI approaching 1.0 describes completely aligned and similar strain curves and shows the entirety of the heart contracts and relaxes together while a SI of 0.0 describes substantial discoordination of myocardial fiber contraction and relaxation throughout the heart.

Standard deviation of time to peak strain (SD-TPS) is a published metric that describes the variability in time from the ECG gated R-wave to peak end-systolic strain (TPS) across all longitudinal and circumferential segments. [21] [22] [23] [24] A SD-TPS less than 50 msec describes closely aligned peak end-systolic strain across all longitudinal and circumferential segments of the heart while SD-TPS greater than 100 msec describes substantial discoordination of segmental longitudinal and circumferential myocardial fiber contraction.

Supplemental Figures S2A and B show the relationship for timing metrics (SI and SD-TPS respectively) at baseline for the incidence of sub-CTX and CTX anytime during follow-up, and at follow-up visits by CTX status. SI and SD-TPS were worse at baseline, pre-treatment for patients who developed sub-CTX and CTX. Patients who developed CTX during cancer treatment had worse markers of mechanical discoordination which were statistically significant but improved during REC. Supplemental Figures S3A and B show the corresponding ROC curves for SI and SD-TPS at baseline for prediction of CTX and during follow-up for the detection of CTX status. The AUCs for SI and SD-TPS were 0.63 and 0.61 for prediction of CTX, and 0.75 and 0.74 for detection of CTX status demonstrating good accuracy in identifying CTX.

Supplemental Figure S3 (A and B). ROC Curves of F-SENC Timing Metrics (SI & SD-TPS) for CTX Prediction (A) and CTX Detection (B)

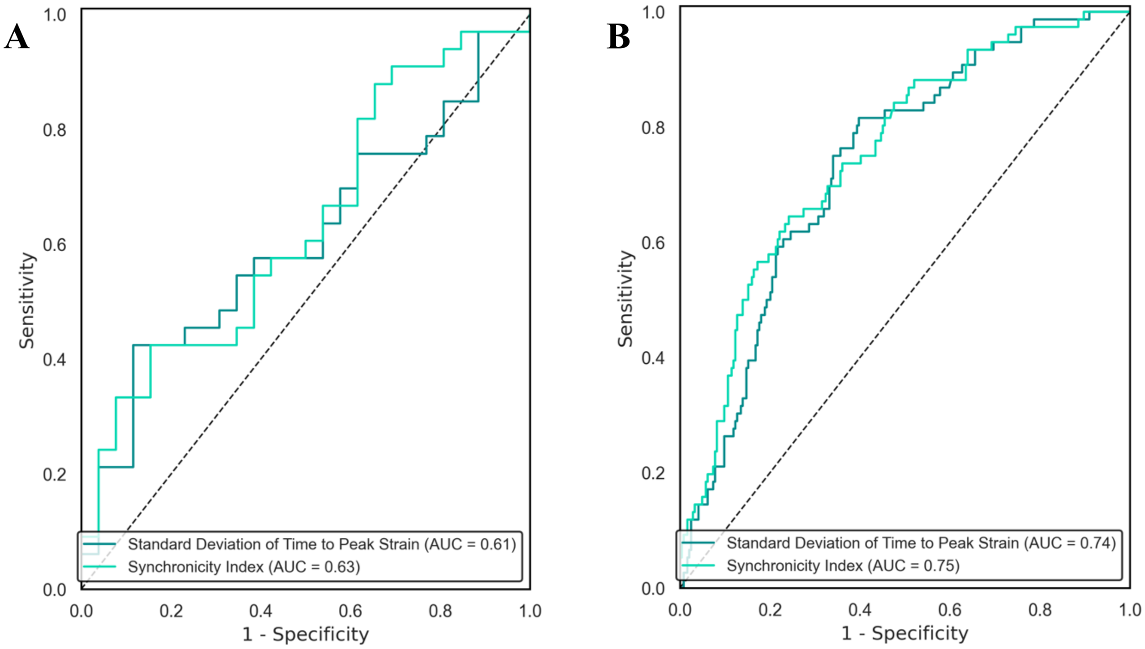

Bibliography

[1] S. Giusca, G. Korosoglou, M. Montenbruck, B. Gersak, A. K. Schwarz, S. Esch, S. Kelle, P. Wulfing, S. Dent, D. Lenihan and H. Steen, "Multiparametric early detection and prediction of cardiotoxicity using myocardial strain, T1 and T2 mapping, and biochemical markers: a longitudinal cardiac resonance imaging study during 2 years of follow-up," *Circulation Cardiovascular Imaging,* vol. 14, p. e012459, 2021.

[2] B. D. Rosen, V. R. Fernandes, K. Nasir, T. Helle-Valle, M. Jerosch-Herold, D. A. Bluemke and J. A. Lima, "Age, increased left ventricular mass, and lower regional myocardial perfusion are related to greater extent of myocardial dyssynchrony in asymptomatic individuals. The Multi-Ethnic Study of Atherosclerosis," *Circulation,* vol. 120, no. 10, pp. 859-866, 8 September 2009.

[3] R. K. Sharma, S. Donekal, B. D. Rosen, M. C. Tattersall, G. Volpe, B. Ambale-Venkatesh, K. Nasir, C. O. Wu, J. F. Polak, C. E. Korcarz, J. H. Stein, J. Carr, K. E. Watson, D. A. Bluemke and J. Lima, "Association of subclinical atherosclerosis using carotid intima-media thickness, carotid plaque, and coronary calcium score with left ventricular dyssynchrony: the Multi-Ethnic Study of Atherosclerosis," *Atherosclerosis,* vol. 239, no. 2, pp. 412-418, April 2015.

[4] R. K. Sharma, G. Volpe, B. D. Rosen, B. Ambale-Venkatesh, S. Donekal, V. Fernandes, C. O. Wu, J. Carr, D. A. Bluemke and J. A. Lima, "Prognostic implications of left ventricular dyssynchrony for major adverse cardiovascular events in asymptomatic women and men: the Multi-Ethnic Study of Atherosclerosis," *Journal of the American Heart Association,* vol. 3, p. e000975, 2014.

[5] A. Zweerink, W. M. van Everdingen, R. Nijveldt, O. A. Salden, M. Meine, A. H. Maass, K. Vernooy, F. J. de Lange, M. A. Vos, P. Croisille, P. Clarysse, B. Geelhoed, M. Rienstra and v. Gelder, "Strain imaging to predict response to cardiac resynchronization therapy: a systematic comparison of strain parameters using multiple imaging techniques," *ESC Heart Failure,* vol. 5, pp. 1130-1140, 2018.
